# College and Hospital Notes

**Published:** 1900-02

**Authors:** 


### COLLEGE AND HOSPITAL NOTES.

---

THE Buffalo Hospital of the Sisters of Charity has received through the Hon. Wilson S. Bissell $7,000 bequeathed to that institution by the late George Howard Lewis. This early settlement of this bequest was made at the request of Mrs. Lewis, in order that the hospital should receive the money during the holiday season, as it was Mr. Lewis's custom to remember it in the annual distribution of his Christmas charities.

---

St. Vincent's Hospital, New York, has received an electric ambulance, which was given by Edward Kelly, the son of Eugene Kelly, the banker, to which allusion was made in our October edition. The ambulance is ready for use and is equipped with windows in front and rear and can be made as warm and comfortable in cold weather as in the mild seasons. It is estimated that it can make easily ten miles an hour.

---

The New York Post-Graduate Hospital has issued its fifteenth annual report, which is for the year ending October 1, 1899. This hospital furnishes unexcelled facilities for the study of disease to the matriculates of the Post-graduate Medical School, which is its primary object. It has a well-equipped laboratory for pathological, chemical and bacteriological study, and it also has a large dispensary where deserving poor are treated as out-patients. Connected with the institution are 112 physicians and surgeons who serve in the wards and teach in the school.

During the past year 523 practitioners of medicine from 57 states and countries have attended the school. There is a babies ward of 53 beds which are absolutely free, and are utilised for clinical instruction. An orthopedic ward is also attached, containing 16 free beds. Besides the usual wards for men and women there is a lying-in department connected with the out-patient branch. These women are served at any time, day or night, during the parturient period, often in homes of utter poverty and with squalid surroundings. A lying-in ward at the hospital is greatly needed and an appeal is made for that purpose.

A summary of the work of the hospital shows as follows: Number of patients treated in the hospital during the year, 2,668; number treated at the dispensary, 17,454; number of confinements attended by house physician, 368. Amount of money received from students' fees and certificates and given to the hospital, $30,046.00. None of the physicians or surgeons receive a salary. The total cost of maintenance of the hospital was $83,780.26; of babies' wards, $14,479.97.

We glean these data from the report which is a model of exactness and detail, illustrated with numerous half-tone engravings, (among which is one of the Margaret Fahnestock Training School for Nurses, which is attached to the hospital,) and bespeaks of a noble work that is conducted on a large scale at a minimum of cost.

